# Comment on Dinesen et al. Diphoterine for Chemical Burns of the Skin: A Systematic Review. *Eur. Burn J.* 2023, *4*, 55–68

**DOI:** 10.3390/ebj4020023

**Published:** 2023-06-15

**Authors:** Alan Hall, Amal Bouraoui, Karine Padois, Joel Blomet, Denise Jacquemin, François Burgher, Lucien Bodson, Jean-Luc Fortin, Howard Maibach

**Affiliations:** 1 Toxicology Consulting and Medical Translating Services, Azle, TX 76020, USA; 2 Laboratoire Prevor, 95760 Valmondois, France; 3 Burns Center, University Hospital, 4000 Liege, Belgium; 4 Emergency Department, University Hospital, 4000 Liege, Belgium; 5 Emergency Department, University Hospital, 25000 Besançon, France; 6 Department of Dermatology, University of California-San Francisco, San Francisco, CA 94143, USA

We read with interest the recent publication of Dinesen et al. [1] regarding a systematic review on employing DIPHOTERINE^®^ solution (Laboratoire Prevor, Valmondois, France) for decontamination of chemical skin splashes. The authors concluded that in comparison with water rinsing or without water rinsing, DIPHOTERINE^®^ solution was associated with less pain and a more rapid return of skin pH to the physiological tolerable range. In this systematic review, rinsing with DIPHOTERINE^®^ solution was not associated with decreased injury depth.

Based on the finding that DIPHOTERINE^®^ solution rinsing had no effect on skin injury depth, it can be interpreted that the injury had already been established by the time the rinsing was performed. In fact, neither potable water nor DIPHOTERINE^®^ solution are treatments for chemical skin injuries. They both act as flushing solutions that aim to mechanically remove the chemical from the skin surface. Flushing solutions do not have an effect on an already existing injury, which explains the importance of intervention time during the emergency management of a chemical splash in order to prevent injury development. Due to its hypertonic, amphoteric, and chelating properties, DIPHOTERINE^®^ solution can extract the chemical that has penetrated but has yet to react with the tissues, which might prevent progression of further injury depth.

We acknowledge and appreciate that Dinesen et al. [1] meticulously followed all applicable guidelines for performing systematic reviews; we question whether the dataset to which they were applied was appropriate for this type of analysis. Of 540 non-duplicate papers, only 55 were selected for full-text review and only 9 were analyzed (~1.6%). Dinesen et al. [1] stated that the nine papers analyzed had “a low certainty of evidence”.

All observational studies, case series, case reports, and preclinical studies were excluded by the authors. In our opinion, these data might have been highly valuable to their review, particularly in the absence of and difficulties in performing blinded controlled clinical studies. Alexander et al. [2] reviewed preclinical and clinical studies and found that DIPHOTERINE^®^ solution appeared to be safe and was probably superior to other flushing fluids. Although noting potential biases regarding lack of randomization or double-blind clinical studies, they affirmed that based on existing evidence, DIPHOTERINE^®^ solution showed reduced tissue necrosis, severity of symptoms, a more rapid return to a physiologically tolerable pH, and decreased pain [2].

There are currently more than 127 million chemical substances [3], and chemical skin injuries account for only 3 to 5% of all burns [4]. There are therefore significant practical and ethical difficulties in comparing flushing fluids for chemical splashes. It is clear that the more rapidly the intervention is performed, the greater the likelihood of better outcomes. This leaves little time to obtain informed consent for double-blind studies. If a flushing fluid is available, it is unethical to have a no rinsing control group, especially when corrosive substances that might have significant or fatal systemic toxicity are involved.

A good alternative would be conducting preclinical, ex vivo, and in vitro studies in order to compare rinsing solution effectiveness. Such experimental results can then be compared to existing clinical data and case reports in order to confirm or reject the preclinical results.

Cavallini et al. [5] studied the effect of several rinsing solutions on blood concentrations of β-endorphin and substance P and wound healing in rats contaminated with hydrochloric acid. The results indicated that the best systemic inflammatory response reduction and best wound healing was from using DIPHOTERINE^®^ solution compared to the controls. Such results have been corroborated by clinical cohorts such as Fortin et al. [6] and Viala et al. [7], which showed a decrease in pain after DIPHOTERINE^®^ solution rinsing.

Rihawi et al. [8] studied the pH in the anterior chamber after applying sodium hydroxide solution on rabbit eyes. The results indicated that DIPHOTERINE^®^ solution led to a significant decrease in pH compared to the controls (a water and saline solution). These findings were confirmed by clinical studies published by Zach Williams et al. [9] where a significant pH change before and after DIPHOTERINE^®^ solution rinsing was observed.

Moreover, even in the case of an equivalent clinical outcome between water and DIPHOTERINE^®^ solution, there are potential beneficial operational advantages to utilizing DIPHOTERINE^®^ solution as a flushing fluid for chemical splashes. All commercialized forms of this solution for chemical skin splash decontamination are “man-portable” in the form of 100 ml, 200 mL, or 5 L spray containers. Therefore, there is no need for expensive installation or maintenance costs of plumbed equipment. Compared to the potable water volumes recommended by the European Norm or the American Standard (ANSI/ISEA Standard), the volumes required for efficacious decontamination with DIPHOTERINE^®^ solution are very much smaller and require much less time for application. This mitigates the need for wastewater management. The risk of hypothermia for the chemically splashed patient is also considerably reduced. These operational advantages are a further indicator of the superiority of DIPHOTERINE^®^ solution use after a chemical splash when intervention time and a thorough rinsing are key to the prevention or mitigation of lesion development.
